# Dynamic changes in the hippocampal memory index and biochemical indices in *Sprague Dawley* rats exposed to intrauterine kola nut

**DOI:** 10.1007/s11011-024-01423-7

**Published:** 2024-11-15

**Authors:** Foluso A. Atiba, Pilani Nkomozepi, Felix E. Mbajiorgu, Amadi O. Ihunwo

**Affiliations:** 1 School of Anatomical Sciences, Faculty of Health Sciences, University of the Witwatersrand, Johannesburg, South Africa; 2 Department of Anatomy, College of Medicine, University of Ibadan, Ibadan, Nigeria; 3 Department of Human Anatomy and Physiology, University of Johannesburg, Doornfontein Campus, Johannesburg, South Africa

**Keywords:** Behavior, Kola nut, BDNF, Malondialdehyde, Acetylcholine

## Abstract

Kola nut is commonly consumed by pregnant women to suppress symptoms of morning sickness. This study investigated the effects of kola nut on the biochemical indices of the hippocampus and its dependent memory. Kola nut extract was fed to pregnant dams from the first day of their pregnancy until parturition. The following behavioral function tests were conducted: surface righting (SR); cliff avoidance (post-natal day [PND] 4, 5, 6 & 7); open field; novel object recognition and location; and radial-arm maze (PND 21 and 56). The levels of brain-derived neurotrophic factor (BDNF), acetylcholine (ACh), and malondialdehyde (MDA) of the matched hippocampal tissues were also checked in the pups. The kola nut-treated pups showed significantly reduced behavioral indices compared to the pups in the control group: lower postural balance, higher risk avoidance memory, and lower frequency in pivoting and rearing compared to that in the control group. However, the frequency of urine and fecal bolus was significantly lower in the pups in the control group than that in the treated pups. The discrimination ratio of the control group pups in novel object recognition (NOR) and novel object location (NOL) was significantly higher than that in the treated pups, and the time taken by the treated pups to complete RAM was significantly higher. The levels of ACh and BDNF in the treated pups were increased compared to that in control pups. A positive correlation was found between MDA and SR (r = 0.7207; *p* = 0.0437), grooming (r = 0.7707; *p* = 0.0252), and fecal bolus (r = 0.7606; *p* = 0.0284), as well as with the BDNF level in those treated with grooming (r = 0.7570; *p* = 0.0297). However, negative correlations between ACh and rearing (r = -0.8261; *p* = 0.0115) and fecal bolus (r = -0.8066; *p* = 0.0156) and a positive correlation with NOL (r = 0.8358; *p* = 0.0098) were observed. Based on these observations, the study concluded that Kola nut affects both biochemical and hippocampal memory profiles.

## Introduction

The hippocampal contributions to flexible cognition are perhaps the most apparent in the complex dynamics of learning, memory, and social interactions. This flexible cognition is usually considered in the context of the executive functions that support the ability to contribute to high-level behavior, such as planning, organizing, and decision-making (Rubin et al. 2014; Palombo et al. 2019). Behavior has always been a major part of animals’ and human beings’ way of living, and cognitive evaluations are an integral part of neuroscience research (Baj et al. 2012; Owoeye et al. 2019). Two major aspects of cognitive evaluations are learning and memory. Learning can be evaluated by conducting repeated training trials to measure acquisition, and memory can be repeatedly assessed across time to evaluate retention (Moser 2011). Different factors have been implicated in causing adverse effects on the ability to learn and reproduce what was learned after a given time, that is, the consolidation of what was learned. Some of these factors stem from teratogens that cause body toxicity, especially in the highly sensitive brain (Atiba et al. 2021), implicating a shift or alteration in the biochemical, structural, and behavioral indices of the brain.

Developing brains are prone to toxic substances that pregnant women consume in ignorance, and these can be passed from the mother to the fetus through the placenta during pregnancy. Some of the substances that pregnant women consume are medications prescribed by medical personnel, but most are self-prescribed as an alternative to orthodox medications, such as herbal concoctions, which are majorly passed down through generations (Onyiapat et al. 2011; Atiba et al. 2023). Most of these substances which contain caffein are consumed to suppress symptoms of morning sickness associated with pregnancy, such as nausea and vomiting (Nyango et al. 2012; Atiba et al. 2023). Studies on human and animal subjects revealed that exposure to harmful substances provokes maternal redo-inflammatory mediators. This delays myelinogenesis and neurogenesis and, thereby, reduce fetal brain growth, particularly in the areas of the hippocampus (Whitelaw and Thoresen 2000; Ben Mimouna et al. 2018; Pintican et al. 2019). Consequently, the effects are highly notable after birth and during the child’s growth (Pintican et al. 2019).

Kola nut is one such substance consumed by pregnant women in sub-Saharan Africa, especially Nigeria. The kola plant is an important plant widely utilized in Africa, and the kola nuts are especially important. Kola nuts belong to the family of Malvaceae, and *Cola acuminata* and *Cola nitida* species are popularly grown in the tropical regions of Africa (Tachie-Obeng and Brown 2004; Atanda et al. 2011). In some parts of Africa—Nigeria, for instance—these nuts are used in ceremonial gatherings during traditional rites and in ethnomedicine (Tachie-Obeng and Brown 2004). Traditionally, fresh seeds are used in the treatment of many ailments, including infections, toothaches, morning sickness, skin diseases, and ulcers (Tachie-Obeng and Brown 2004). It is also known as a stimulant and appetite suppressant (Ojo et al. 2010). Fresh nuts are used in the commercial production of beverages, especially cola drinks, wines, and chocolates (Ogutuga 1975; Dah-Nouvlessounon et al. 2015).

The pharmacologically active components of *Cola nitida* include caffeine, theobromine, catechins, and tannins (Burdock et al. 2009; Starin 2013; Nyadanu et al. 2020). Pregnant women especially depend on the anti-nausea effect of this rich nut as it has been shown to repress the occurrence of morning sickness during pregnancy (Nyango et al. 2012; Atiba et al. 2023). The nut has been shown to possess antioxidant, apoptotic, anti-inflammatory, anti-microbial, anti-diabetic, and cardioprotective effects amongst others (Tachie-Obeng and Brown 2004). Studies on the toxicological properties and effects of kola nuts have been reported (Ikegwuonu et al. 1981; Burdock et al. 2009; Atiba et al. 2021), and implicated in organ toxicity (Nneli et al. 2007; Umoren et al. 2011; Emmanuel et al. 2016). Its caffeine crosses the placenta, penetrates the blastocyst germ layer (Fabro and Siever 1969), and accumulate in the fetal brain (Galli et al. 1975; Tanaka and Nakazawa 1990; Atiba et al. 2021). As a significant number of studies have reported the beneficial effect of kola nuts, it remains undiscovered whether this plant product exhibits positive or negative gestational effects on pups’ hippocampi after birth, when consumed by pregnant mothers. Additionally, the potential mechanism of action involved in these effects on hippocampal function (spatial learning/memory) and its modulators (brain-derived neurotrophic factor [BDNF] and acetylcholine [ACh]) await discovery.

## Methodology

### Kola nut filtrate extraction

Fresh red (commonly consumed) kola nut fruits were procured from the plantation of Ode Remo, Shagamu, Ogun state, Nigeria, and authenticated by a botanist at the Federal Research Institute of Nigeria (Voucher number or Federal Herbarium Index number 109605). The average weight (4.5 kg) of kola nuts were thoroughly washed to remove dirt, cut into small cubes, and dried at room temperature until a constant weight was achieved (1 kg). The dried kola nuts were ground with a miller and mixed with distilled water. The soaked and milled kola nuts were hourly vortexed for six hours, and the supernatant was filtered using a Whatman filter paper number 1 (cat. no. 1001. 125). The filtrate was concentrated using a rotary evaporator (BUCHI Labortechnik AG: CH-9230 Flawil Switzerland), freeze-dried (LTE Lyotrap plus by LTE scientific LTD. Great Britain), and stored in an airtight container in the refrigerator at 4 °C.

### Animal treatment

Twelve dams were randomly selected and equally allocated to two groups: the control group and the treated group. The dams in the treated group received gelatin infused with 400 mg/kg body weight of the aqueous crude extract of the kola nuts (Atiba et al. 2021) from the first day of their pregnancy until parturition, while the dams in the control group received plain gelatin without the kola nut extract. The dams were maintained under sterile conditions in individual Perspex cages 43 mm long, 220 mm wide, and 200 mm high, allowing the free movement of the dams. After the parturition, the pups remained with their biologic dams until they were weaned on post-natal day (PND) 21, then they were sorted into individual cages. Standard animal house conditions were maintained (temperature: 25^0^C; light alternated in approximately 12 h’ light–dark cycle). Twenty pups were randomly selected from the treated and the control groups with ten pups in each.

### Ethics

All the procedures on animal handling conformed to the acceptable guidelines on the ethical use of animals in research as approved by the Animal Research Ethics Committee of the University of the Witwatersrand, Johannesburg (Ethical clearance certificate number: 2020/06/02/C).

### Behavioral tests

The cognitive tests to assess hippocampal function were cliff avoidance (CA), surface righting (SR), spontaneous movements, open field, object recognition and location, and radial-arm maze (RAM) test. On PND 4, 5, 6, and 7, 10 animals from each group underwent CA, SR, and spontaneous movements; while open field, novel object recognition, and location and RAM were conducted only on PND 21 and 56 to measure the kola nuts’ effects on short- and long-term memory. All the tests were conducted between 9:00 am and 3:00 pm (Fujimoto et al. 2015), and the animals were brought into the test room 30 min before the actual tests for acclimatization (Denninger et al. 2018). The test apparatus was cleaned with 70% alcohol in between tests to prevent odor cues for the subsequent test animal.

### Cliff avoidance

Following gestational exposure to kola nut consumption, a CA test was conducted to assess the developmental reflex and memory in neonates (post-birth). The time taken for each animal to turn from the cliff was recorded. The decrease in the time over four days was used to measure learning and memory. A combination of the methods used by Tanaka and Nguyen et al. was employed (Tanaka 2012; Nguyen et al. 2017). This test was done on PND 4, 5, 6, and 7 on pups of both the control and the treated groups.

### Surface righting

This test was used to assess the developmental reflex and memories of the pups on PND 4, 5, 6, and 7, through the repetition of the test for four days. The decrease in the time taken to turn from their backs unto their paws was used to assess learning and memory (Nguyen et al. 2017).

### Spontaneous movements

The locomotor activity of the pups on PND 4, 5, 6, and 7 was assessed using the spontaneous movement test model. The test was conducted in a glass Perspex container of 10 cm in diameter and 10 cm in height, instead of 5 cm in diameter and 10 cm in height, as done by Tartaglione et al. (2020), for easy placement and removal of the animals. All other methods were followed. Different measurements were conducted within the speculated time of 2 min, based on the methods employed by Tartaglione et al. (2020). This includes locomotion, head raising, and pivoting. The frequency and duration of each behavioral pattern were checked and recorded (Tartaglione et al. 2020).

### Open field

This test was used to assess locomotor activity, exploratory movements, and anxiety in the pups at PND 21 and 56. Each animal was placed in a 120 cm × 120 cm box with a black background for 5 min. The open field box was cleaned with 70% ethanol and allowed to dry between tests. The distance traveled by each animal and the mean velocity were recorded. The line crossing, as well as the number of freezing, rearing, and grooming episodes, were also recorded and analyzed with ANY-maze behavioral tracking software (Tartaglione et al. 2020). The presence of fecal bolus and frequency of urination was noted.

### Novel object recognition and location (NOR/NOL)

The NOR/NOL tasks were used to evaluate the impaired cognition in recognizing a novel object and location within an environment without any positive or negative reinforcement. This was based on the methodology of Ennaceur and Delacour (1988) and Gerstein et al. (2013). The following parameters were measured: the total amount of time spent exploring the novel and familiar object (NOR) and at the novel location (NOL), novelty index, and discrimination index. The relative exploration time (novelty index) was calculated using the following formula:

$$\begin{array}{l}\text{Novelty index}=\\\frac{\text{Time spent exploring the object in a novel location}}{\text{Time spent exploring both objects in total}}\times100\end{array}$$

The discrimination index was calculated using (tn—tf)/(tn + tf),

where tn refers to the amount of time for which the rats explored the novel object and tf is the amount of time for which the rats explored the familiar object.

### Radial- Arm Maze (RAM)

RAM was used to test for spatial memory in the pups in both the groups on PND 21 and 56. The test consisted of three stages. The first was habituation, in which each animal was placed in the center and allowed to freely roam for 5 min within the maze for 3 consecutive days with daily reward snacks (but reduced on the last day of habituation) scattered at the center and arms of the maze. The habituation stage ended when all the arms had been visited. The following two stages, the training, and the test stages, were conducted on the same day for 4 consecutive days. The modified apparatus consisted of eight horizontal arms (57 cm × 11 cm) placed radially away from a central region above the floor and the doors (20 cm high) placed at the entrance of each arm. The animals were deprived of food during the dark cycle and given the baits daily for 3 days before the test began. This helped to stimulate them to eat the baits during the test (Dale and Roberts 1986; Timberlake and White 1990; De Luca et al. 2016). During the training stage, four of the arms were blocked; the remaining four were opened and baited, and the rats were allowed to freely explore them for 5 min to retrieve the baits. After 5 min of training, the animals were retrieved and kept in their individual cages for 5 min while the arms were wiped with 70% alcohol and dried. The blocked arms were now opened and baited, and each animal was brought to the center and tested for another 5 min. The time taken to finish each task was recorded for both the training and the test periods (Delcourt et al. 2018).

### Termination of the animals

After the behavioral tests for each timeline had been conducted, the animals were anesthetized and sacrificed at different stipulated timelines with sodium pentobarbitone (Bayer [Pty] Ltd. Co. Reg. No. 1968/011192/07, South Africa) at 1 to 2 ml/kg body weight intraperitoneally. The animals were then transcardially perfused with phosphate-buffered saline. The hippocampus from their left hemisphere was quickly dissected on ice, wrapped in industrial foil paper, and stored at -80^0^C until it was ready for biochemical analyses. The hippocampal tissues were homogenized in 0.01 M phosphate-buffered saline over ice using a homogenizer (Eppendorf MiniSpin plus; Cat no: 22620100). Thereafter, the homogenate was centrifuged (Tissue Grinder-G5; Cat no: 2002000301) for 10 min at 10000 rpm, and the upper layer was aspirated into a clean tube using a pipette for biochemical analyses.

## Biochemical determination of lipid peroxidation, brain-derived neurotrophic factor and acetylcholine in the hippocampal homogenates

### Determination of lipid peroxidation (Malondialdehyde - MDA)

The MDA level, a measure of lipid peroxidation activity in the animal hippocampus, was measured according to (Sathishkumar et al. 2010). This was to determine the effects of kolanut on oxidative process/stress and its possible mechanism of action on the cognitive process of brains exposed to kolanut. MDA is recognized as the commonly used marker for oxidative stress and used on that basis. The tissue supernatant aliquots (100 μL) were mixed with Tris-KCl buffer (900 μL), and 30% TCA (500 μL) was then added. For 45 min at 80 °C, 500 μL of thiobarbituric acid (0.75%) was further added and heated using a water bath. Thereafter, the mixture was centrifuged at 3000 rpm g for another 5 min, and the absorbance was read at 532 nm. The MDA formed was calculated by taking the molar extinction coefficient of 1.56 × 105/M/cm, expressed as nmol MDA mg − 1 protein.

### Determination of Brain derived neurotrophic factor (BDNF)

The determination of BDNF was done to ascertain its level after the administration of kolanut. It was carried out using an ELISA kit (Elabscience® Catalog No: E-EL-R1235, USA), which was based on a sandwich-ELISA technique. The micro-ELISA plate provided in the kit was precoated with an antibody specific to rat BDNF. The color change was measured by a Biochrom Anthos 2010 microplate reader at a wavelength of 450 nm. The BDNF concentration in the samples was then determined as per the manufacturer’s instructions.

### Determination of Acetylcholine (ACh)

The level of Ach was determined after ingestion of kolanut to correlate its level with memory accumulation and consolidation of the pups. This was carried out using an ELISA kit (Elabscience® Catalog No: E-EL-0081, USA), based on a competitive-ELISA principle. The ACh in the samples and standard competed with the fixed amount of ACh on a solid phase supporter for sites on the Biotinylated Detection Ab specific to ACh. The color change was measured at a wavelength of 450 nm by the Biochrom Anthos 2010 microplate reader. The concentration of ACh in the samples was then determined as per the manufacturer’s instructions.

### Statistical analysis

The data were expressed as mean ± standard error of the mean (SEM). Shapiro-Wilks test was conducted to determine the normalcy of the data, and a two-way analysis of variance was done followed by the Bonferroni *post-hoc* test. The association between the behavioral tests and the biochemical analyses was investigated using logistic regression analysis. Statistical analyses were conducted using GraphPad Prism (version 9.0). A *p* value < 0.05 was considered significant.

## Results

### Cliff avoidance test

**Fig. 1:**
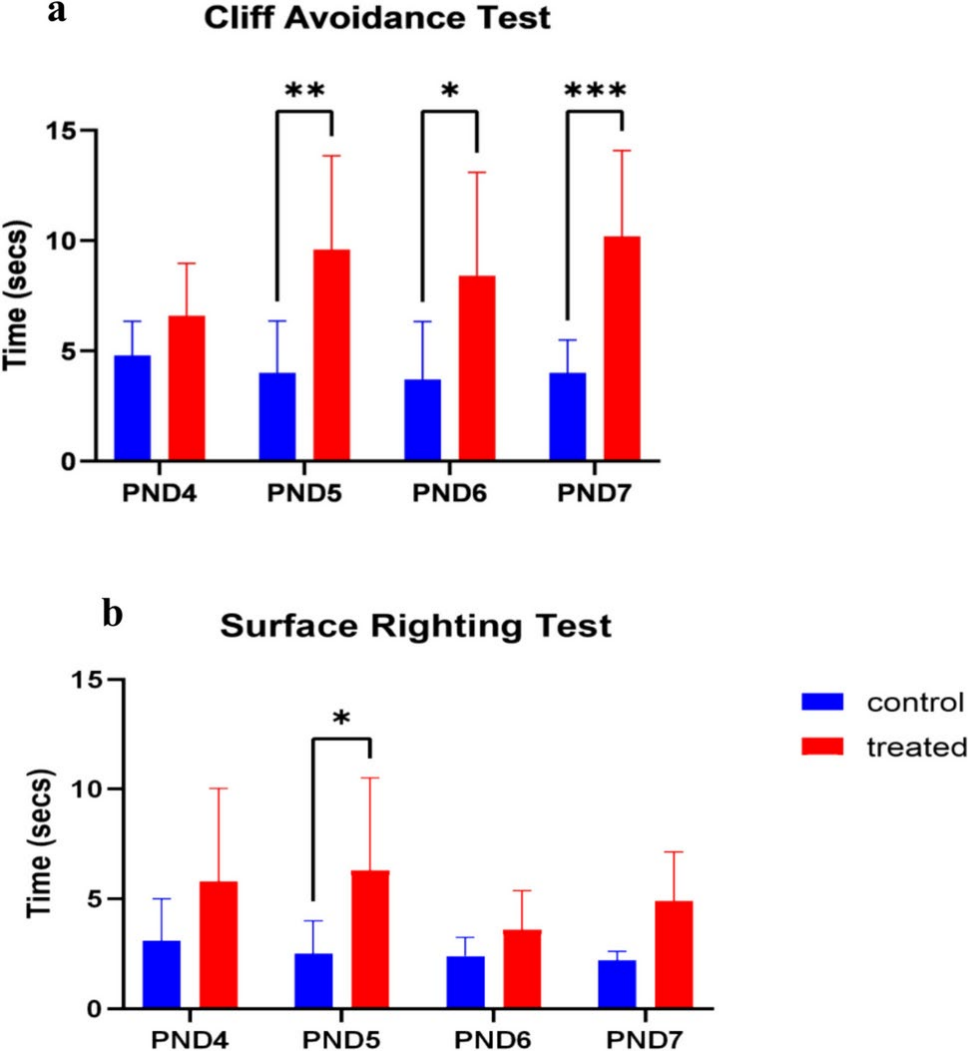
Effects of kola nut exposure on cliff avoidance test (**a**) and surface righting test (**b**). The diagrams show the results of the cliff avoidance and surface righting tests in secs in the pups from the kola nut-treated dams on PND 4, 5, 6 and 7. The data are represented by mean ± SE with 10 pups in each group. The values *p < 0.01, ** *p* < 0.001, and ***p < 0.0001 indicate significant differences between the two groups

CA was used to compare the learning and memory of the pups prenatally exposed to kola nut extract to that of the pups in the control group. The avoidance of the prenatally exposed pups took a longer time than that of the pups in the control group at all ages. CA was significant at different treatments, mean effect of treatment (F _[1,72] =43.03_; *p* < 0.0001), the mean effect of age (F _[3,72] =0.8647_; *p* = 0.4635), and interaction (F _[3,72] =1.954_; *p* = 0.1286) were determined as well (Fig. 1).

### Surface righting test

The latency of the SRT was significantly different on different treatments at PND 5. The mean effect of treatment (F _[1,72] =21.33_; *p* < 0.0001), mean effect of age (F _[3;72] =1.551_; *p* = 0.2087), and interaction (F _[3;72] =1.8992_; *p* = 0.4459). The latency of SRT for the control group gradually decreased as the age increased (Fig. 1).

### Spontaneous movements

#### Head raising

**Fig. 2:**
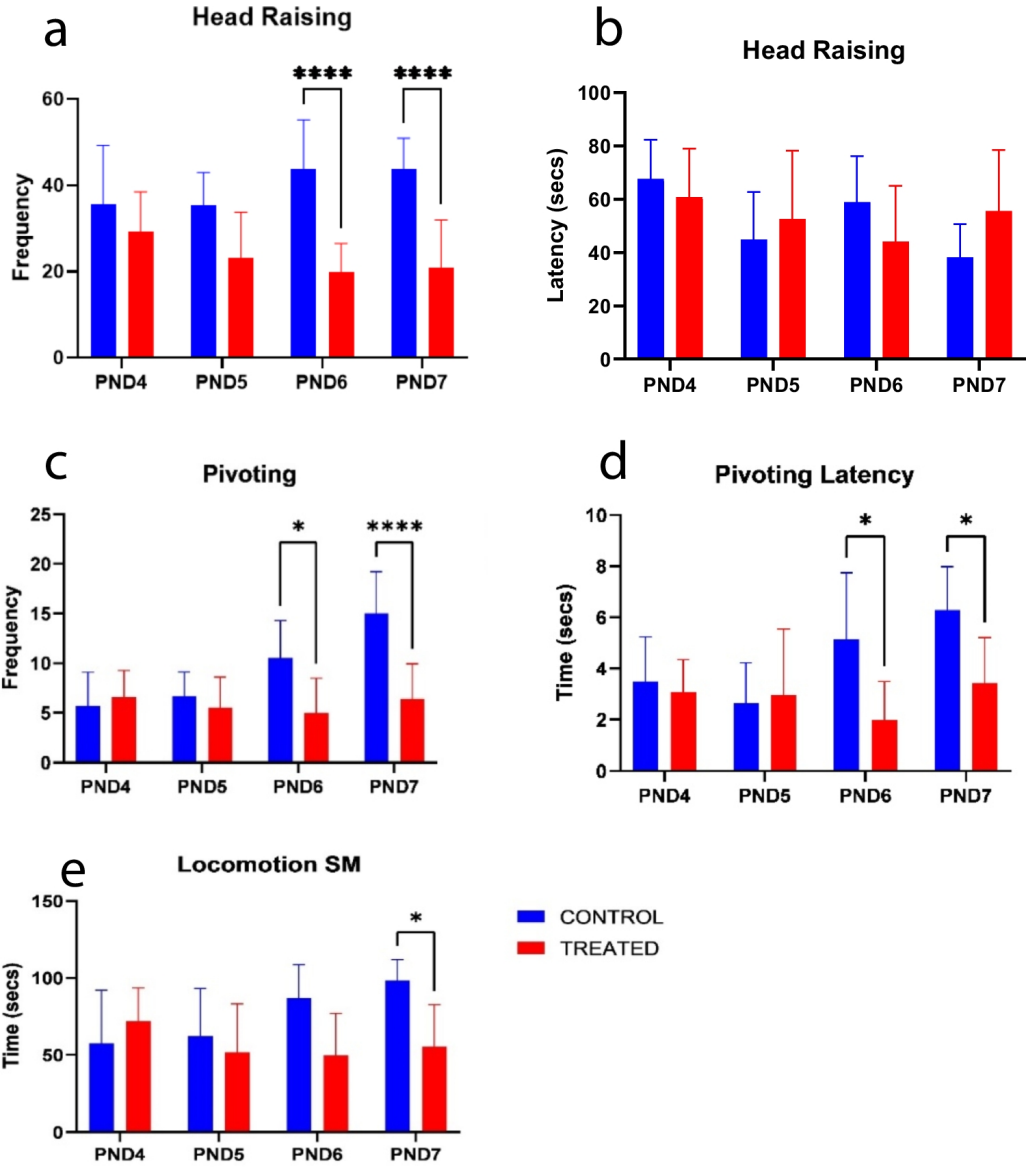
Effects of kola nut exposure on spontaneous movements as seen in head raising tests (**a**, frequency; **b**, latency); pivoting (**c**, frequency; **d**, latency); and locomotion (**e**). The diagrams show results in secs of frequency and the time it took to raise the head in the given time slot (**a**, **b**); results of frequency and latency on pivoting of the head (**c**, **d**); and results of locomotion (**e**) in pups from kola nut-treated dams on PND 4, 5, 6, and 7. The data are represented by mean ± SE, with 10 pups in each group. The values *p < 0.01 and ****p < 0.0001 indicate significant differences between the two groups

The frequency of head raising was significantly different, mean effect of treatment (F _[1,72] =10.05_; *p* = 0.0022), mean effect of age (F _[3,72] =1.789_; *p* = 0.1569); and interaction (F _[3,72] =2.956_; *p* = 0.0381). The latency of head raising was significantly not different at different treatment, mean effect of treatment (F _[1,72] =0.03978_; *p* = 0.88425), mean effect of age (F _[3,72] =3.317_; *p* = 0.0246) and interaction (F _[3,72] =2.801_; *p* = 0.0460) (Fig. 2).

#### Pivoting

The treatment given across the age groups differed significantly on the frequency of pivoting, as shown by the mean effect of treatment (F _[3,72] =22.81_; *p* < 0.0001), the mean effect of age (F _[3,72] =8.193_; *p* < 0.0001) and interaction between treatment and age (F _[3,72] =8.013_; *p* = 0.0001). The number of pivoting times for the control group increased every day with the highest on PND 7 while that of the treated group decreased as the days increased. The treatment given across the age groups also significantly differed, as shown by the mean effect of treatment (F _[1,72] =12.88_; *p* = 0.0006), mean effect of age (F _[3,72] =8.193_; *p* = 0.0075), and a significant difference in the interaction between treatment and age (F _[3,72] =8.013_; *p* = 0.0083) (Fig. 2).

#### Locomotion

The treatments given significantly differed in the amount of movement done by kola nut treated pups as compared to the control, mean effect of treatment (F _[1,72] =10.12_; *p* < 0.0022), mean effect of age (F _[3;72] =1.883)_; *p* = 0.1401) and interaction (F _[3,72] =4.872_; *p* = 0.0038) (Fig. 2).

### Open field

The pups were tested in the open field apparatus for different tests at PND 21 and 56.

#### Line crossing

**Fig. 3:**
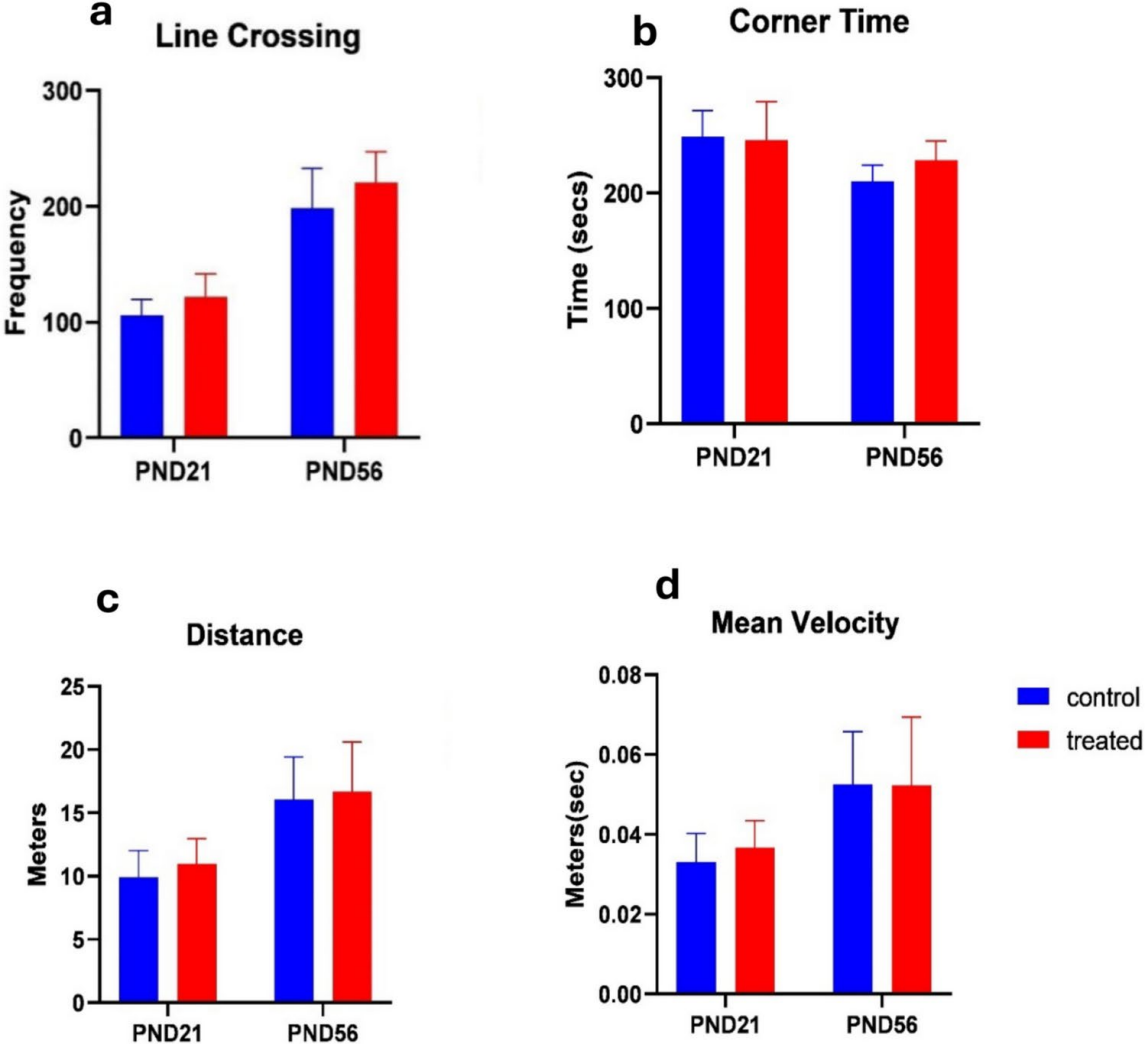
Effects of kola nut exposure on the amount of movement in open field test as seen in line crossing (**a**), corner time (**b**), distance (**c**), and mean velocity (**d**) by the animals. The diagram shows the results of the number of times the animals crossed both vertical and horizontal lines (**a**), the time spent at each corner of the box (**b**), the distance covered (**c**), and the rate of movement in the given time (**d**) of the pups from the kola nut-treated dam between PND 21 and 56. The data is represented by mean ± SE with 10 animals in each group

A significant difference was observed in the treatment given across all age groups in the frequency of line crossing, as shown by the mean effect of treatment (F _[1,36] =5.777_; *p* = 0.0215) and the mean effect of age (F _[1,36] =146.9_; *p* < 0.0001) and interaction (F _[1,36] =0.1743_; *p* = 0.6788) (Fig. 3).

#### Corner time

The time spent at the corner box in the open field test across the age groups significantly differed, as shown by the mean effect of age (F _[1,36] =14.95_; *p* = 0.0004), mean effect of treatment (F _[1,36] =1.118_; *p* = 0.2975) and interaction (F _[1,36] =2.137_; *p* = 0.1525) (Fig. 3).

#### Distance

The distance covered by the pups across the age groups demonstrated a highly significant difference, as shown by the mean effect of age (F _[1,36] =40.51_; *p* < 0.0001), mean effect of treatment (F _[1,36] =0.8567_; *p* = 0.3608), and interaction (F _[1,36] =0.05829_; *p* = 0.8106) (Fig. 3).

#### Mean velocity

The velocity at which the distance was covered at all ages showed a highly significant difference, as shown by the mean effect of age (F _[1,36] =21.88_; *p* < 0.0001), mean effect of treatment (F _[1,36] =0.2176_; *p* = 0.6437) and interaction (F _[1,36] =0.2702_; *p* = 0.6064) (Fig. 3).

#### Freezing episodes

The frequency of freezing across the age groups significantly differed, as shown by the mean effect of age (F _[1,36] =11.16_; *p* = 0.0027), mean effect of treatment (F _[1,36] =3.708_; *p* = 0.062) and (F _[1,36] =0.3475_; *p* = 0.5592) (Fig. 3).

#### Grooming

**Fig. 4:**
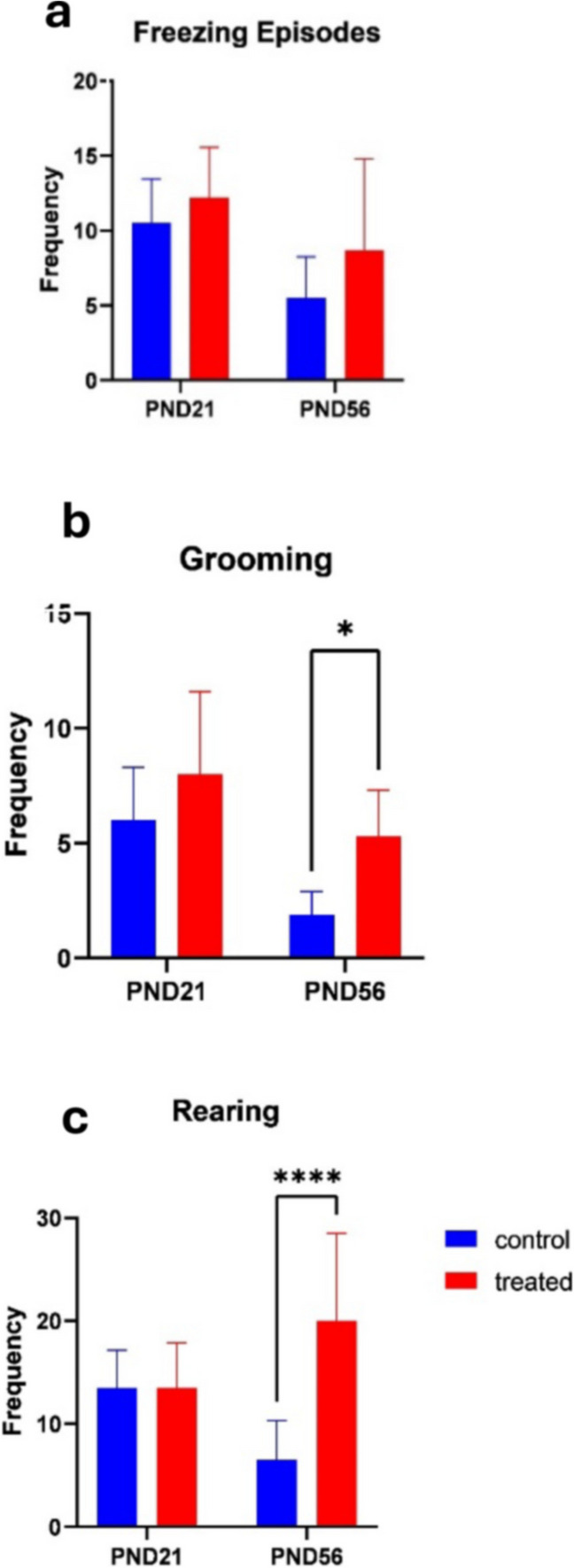
Effects of kola nut exposure on the animals from the open field test on freezing (**a**), grooming (**b**), and rearing (**c**). The diagram shows the results of the number of freezing episodes (**a**), number of grooming times (**b**), and number of times the animals reared (**c**) from the kola nut-treated dam between PND 21 and 56. The data are represented by mean ± SE, with 10 animals in each group. The values *p < 0.01 and ****p < 0.00001 indicate significant differences between the two groups

The frequency of grooming significantly differed with treatments across age groups, mean effects of treatment (F _[1,36] =12.56_; *p* = 0.0011), mean effect of age (F _[1,36] =19.91_; *p* < 0.0001) and interaction (F _[1,36] =3.269_; *p* = 0.8440) (Fig. 4).

#### Rearing

This factor was also used to access memory, especially after the initial contact with the location. The frequency of rearing in the control reduced significantly with different treatments, mean effect of treatment (F _[1,36] =15.28_; *p* = 0.0004), mean effect of age (F _[1,36] =0.02096_; *p* = 0.8857) and interaction (F _[1,36] =15.28_; *p* = 0.0004) (Fig. 4).

#### Fecal bolus and urination

**Fig. 5:**
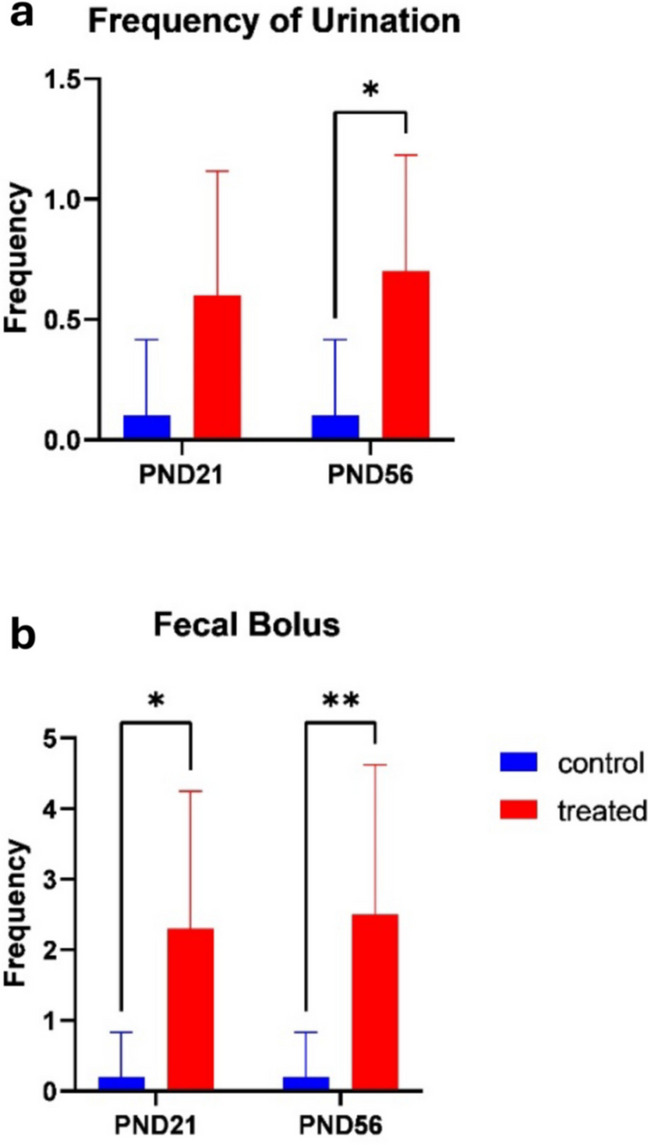
Effects of kola nut exposure in open field test on frequency of urination (**a**) and fecal bolus (**b**). The diagrams show the results of the number of urination (**a**) and fecal bolus (**b**) passed by animals from the kola nut-treated dams between PND 21 and 56. The data are represented by mean ± SE, with 10 animals in each group. The values *p < 0.01 and **p < 0.001 indicate significant differences between the two groups

The number of fecal boli significantly differed with treatments, mean effect of treatment (F _[1,36] =21.30_; *p* < 0.0001), mean effect of age (F _[1,36] =0.04401_; *p* = 0.8350) and interaction (F _[1,36] =0.04401_; *p* = 0.8350). The frequency of urination significantly differed with different treatments and at PND 56, mean effect of treatment (F _[1,36] =17.29_; *p* = 0.0002), mean effect of age (F _[1,36] =0.1429_; *p* = 0.7077), and interaction (F _[1,36] =0.1429_; *p* = 0.7077) (Fig. 5).

#### Novel object recognition

The NOR was conducted on the pups on PND 21 and 56. For PND 21 (Table 1), four out of the ten control (1–10) animals had a positive difference score and discrimination ratio of greater than 0.5, whereas six out of ten (11–20) animals in the treated group had a positive difference score and discrimination ratio. On PND 56 (Table 2), nine control animals had both a positive difference score and a discrimination ratio, while seven animals in the treated group had a positive score and a discrimination ratio. The time spent with the old object was significant across the age groups, mean effect of age (F _[1,36] =2.944_; *p* < 0.0001), mean effect of treatment (F _[1,36] =2.944_; *p* = 0.0948) and interaction (F _[1,36] =4.353_; *p* = 0.0441). The time spent with the new object was also significant across the age groups, mean effect of age (F _[1,36] =90.22_; *p* < 0.0001), mean effects of treatment (F _[1,36] =2.241_; *p* = 0.1431) and interaction (F _[1,36] =4.353_; *p* = 0.1959). The treatment was not significant on novelty index, mean effects of treatment (F _[1,36] =0.3041_; *p* = 0.5847), mean effect of age (F _[1,36] =3.281_; *p* = 0.0784), and interaction (F _[1,36] =0.04545_; *p* = 0.8324).

**Table 1:** Novel Object Recognition on PND 21 showing difference scores and discrimination ratios

S/N	Familiar object time (tf)	Novel object time (tn)	Total time (tn + tf)	Difference score (tn—tf)	Discrimination ratio (tn/total time)
1	17.2	21.8	39.0	4.60*	0.56**
2	37.6	23.4	61.0	-14.20	0.38
3	36.6	25.4	62.0	-11.20	0.41
4	38.3	27.9	66.2	-10.40	0.42
5	19.2	43.3	62.5	24.10*	0.69**
6	26.2	24.3	50.3	-1.90	0.48
7	16.0	7.9	23.9	-8.10	0.33
8	27.5	24.1	51.6	-3.40	0.47
9	0.4	10.1	10.5	9.70*	0.96**
10	28.0	29.2	57.2	1.20*	0.51**
11	5.5	8.1	13.6	2.60*	0.60**
12	13.8	31.5	45.3	17.70*	0.70**
13	47.8	30.2	78.0	-17.60	0.39
14	30.0	5.5	35.5	-24.50	0.15
15	15.3	31.8	47.1	16.50*	0.68**
16	24.4	44.9	69.3	20.50*	0.65**
17	32.5	22.3	54.8	-10.20	0.41
18	14.0	8.5	22.5	-5.50	0.38
19	28.8	32.0	60.8	3.20*	0.53**
20	20.4	23.0	43.4	2.60*	0.53**

1–10 = control pups; 11–20 = treated pups. tn = time spent on a familiar object; tf = time spent on the novel object. * Positive difference score = Object Recognition; ** Discrimination > 0.5 = Object Recognition

**Table 2:** Novel Object Recognition on PND 56 showing difference scores and discrimination ratios

S/N	Familiar object time (tf)	Novel object time (tn)	Total time (tn + tf)	Difference score (tn—tf)	Discrimination ratio (tn/total time)
1	32.1	52.8	84.9	20.70*	0.62**
2	29.4	51.7	81.1	22.30*	0.64**
3	38.5	44.0	82.5	5.50*	0.53**
4	45.3	66.1	111.4	20.80*	0.59**
5	49.0	44.8	93.8	-4.20	0.48
6	44.5	71.9	116.4	27.40*	0.62**
7	35.8	54.0	89.8	18.20*	0.60**
8	40.4	67.4	107.8	27.00*	0.63**
9	22.6	75.7	98.3	53.10*	0.77**
10	37.5	48.8	86.3	11.30*	0.57**
11	78.5	81.8	160.3	3.30*	0.51**
12	49.4	48.6	98.0	-0.80	0.50
13	49.6	76.8	126.4	27.20*	0.61**
14	46.2	50.3	96.5	4.10*	0.52**
15	35.2	74.8	110.0	39.60*	0.68**
16	64.5	62.6	127.1	-1.9	0.49
17	36.3	67.8	104.1	31.50*	0.65**
18	48.5	55.8	104.3	7.30*	0.53**
19	37.4	110.3	147.7	72.90*	0.75**
20	78.3	62.8	141.1	-15.50	0.45

1–10 = control pups; 11–20 = treated pups. tn = time spent on a familiar object; tf = time spent on the novel object. * Positive difference score = Object Recognition; ** Discrimination > 0.5 = Object Recognition

#### Novel object location

The NOL was done on the pups on PND 21 and 56. On PND 21 (Table 3), three out of the control (1–10) pups had a positive difference score and a discrimination ratio of greater than 0.5, and one out of the 10 (11–20) pups in the treated group had a positive difference score and discrimination ratio. On PND 56 (Table 4), four control pups had a positive difference score and discrimination ratio, while only one treated pup had a positive score and discrimination ratio. The time spent at the old location was significant across age groups, mean effect of age (F _[1,36] =9.566_; *p* = 0.0038), mean effects of treatment (F _[1,36] =1.960_; *p* = 0.1701) and interaction (F _[1,36] =0.3093_; *p* = 0.5816). The time spent with the new object was significant across the age groups, mean effect of age (F _[1,36] =88.44_; *p* < 0.0001), mean effects of treatment (F _[1,36] =1.092_; *p* = 0.3030) and interaction (F _[1,36] =0.7331_; *p* = 0.3975). The novelty index was not significant across the age groups, mean effects of treatment (F _[1,36] =1.608_; *p* = 0.2129), mean effect of age (F _[1,36] =0.7776_; *p* = 0.3837), and interaction (F _[1,36] =0.1169_; *p* = 0.7344).

**Table 3:** Novel Object Location on PND 21 showing difference scores and discrimination ratios

S/N	Familiar object time (tf)	Novel object time (tn)	Total time (tn + tf)	Difference score (tn—tf)	Discrimination ratio (tn/total time)
1	26.5	8.8	35.3	-17.70	0.25
2	20.5	29.2	49.7	8.70*	0.59**
3	17.9	24.6	42.5	6.70*	0.58**
4	65.8	23.1	88.9	-42.70	0.26
5	42.0	23.0	65.0	-19.00	0.35
6	7.9	9.8	14.7	1.90*	0.67**
7	19.4	14.7	34.1	-4.70	0.43
8	24.8	13.7	38.5	-11.10	0.36
9	21.1	15.8	36.9	-5.30	0.43
10	16.8	7.1	23.9	-9.70	0.30
11	13.6	10.2	23.8	-3.40	0.43
12	17.2	12.4	29.6	-4.80	0.42
13	26.3	25.7	52.0	-0.60	0.49
14	166.7	16.8	183.5	-149.90	0.09
15	30.6	14.3	44.9	-16.30	0.32
16	16.2	5.8	22.0	-10.40	0.26
17	20.7	3.7	24.4	-17.00	0.15
18	0.4	1.8	2.2	1.40*	0.82**
19	24.1	10.3	34.4	-13.80	0.30
20	26.1	12.4	38.5	-13.70	0.32

1–10 = control pups; 11–20 = treated pups. tn = time spent on a familiar object; tf = time spent on the novel object. * Positive difference score = Object Location; ** Discrimination > 0.5 = Object Location

**Table 4:** Novel Object Location on PND 56 showing difference scores and discrimination ratios

S/N	Familiar object time (tf)	Novel object time (tn)	Total time (tn + tf)	Difference score (tn—tf)	Discrimination ratio (tn/total time)
1	57.2	61.4	118.6	4.20*	0.52**
2	66.3	33.3	99.6	-33.00	0.33
3	63.8	37.5	101.3	-26.30	0.37
4	72.8	56.5	129.3	-16.30	0.44
5	52.0	40.6	92.6	-11.40	0.44
6	45.6	28.5	74.1	-17.10	0.38
7	49.9	25.1	75.0	-24.80	0.33
8	33.5	41.9	75.4	8.40*	0.56**
9	35.2	36.0	71.2	0.80*	0.51**
10	24.5	47.1	71.6	22.60*	0.66**
11	47.2	33.8	81.0	-13.40	0.42
12	61.2	28.2	89.4	-33.00	0.32
13	59.0	49.0	108.0	-10.00	0.45
14	51.3	37.8	89.1	-13.50	0.42
15	41.9	44.9	86.8	3.00*	0.52**
16	131.7	38.0	169.7	-93.70	0.22
17	77.9	49.7	127.6	-28.20	0.39
18	72.5	31.5	104.0	-41.00	0.30
19	95.7	52.7	148.4	-43.00	0.36
20	46.0	36.0	82.0	-10.00	0.44

1–10 = control pups; 11–20 = treated pups. tn = time spent on a familiar object; tf = time spent on the novel object. * Positive difference score = Object Location; ** Discrimination > 0.5 = Object Location

#### Radial-arm maze (RAM)

**Fig. 6:**
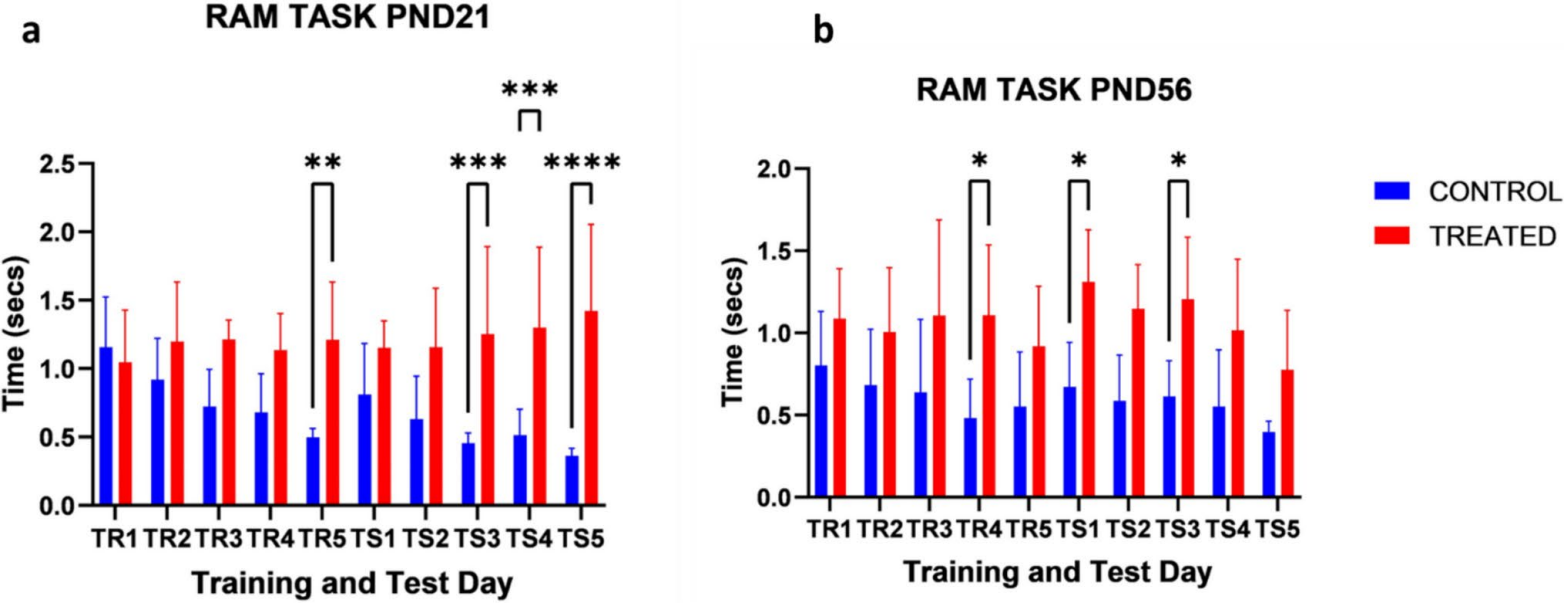
Effects of kola nut exposure on the pups as seen in radial arm maze test on PND 21 (**a**) and PND 56 (**b**). The diagram shows the results of the time taken to complete the task in the pups from the kola nut-treated dams. The data are represented by mean ± SE, with 10 animals in each group. The values *p < 0.01, **p < 0.001, ***p < 0.0001 and ****p < 0.00001 indicate significant differences between the two groups

RAM was conducted on the pups on both PND 21 and PND 56. The times taken to complete the tasks were evaluated. RAM task for PND 21 was significantly different as compared to control, mean effects of treatment (F _[1,180] =107.3_; *p* < 0.0001), mean effects of age (F _[9,180] =1.071_; *p* = 0.3864), and interaction (F _[9,180] =4.048_; *p* < 0.0001). Likewise, the time taken for the completion of the task on PND 56 was significantly higher than control at different treatment groups across the age group, mean effects of treatment (F _[1,180] =90.20_; *p* < 0.0001), age (F _[9,180] =0.7403_; *p* = 0.0250), and interaction (F _[9,180] =0.6823_; *p* = 0.7243) (Fig. 6).

#### TBARS, BDNF, and ACh levels

The MDA levels in the hippocampus of the animals exposed to kola nut across all the age groups was significantly increased compared to their respective controls, as shown by mean effects of treatment (F _[4,30] =4.366_; *p* = 0.0067), mean effects of age (F _[1,30] =12.53_; *p* = 0.0013) and interaction (F _[4,30] =2.406_; *p* = 0.0715) (Table 5).

**Table 5:** Effect of kola nut extract on TBARS, BDNF, and ACh levels markers

PND		0	7	21	56	70
MDA	Control	1.36 ± 0.04	1.20 ± 0.28	1.65 ± 0.15	1.38 ± 0.27	1.27 ± 0.22
	Treated	1.26 ± 0.01*	1.60 ± 0.36*	1.74 ± 0.08*	1.78 ± 0.09*	1.63 ± 0.20*
BDNF	Control	4.11 ± 0.08	4.19 ± 0.21	4.98 ± 0.04	5.31 ± 0.17	4.48 ± 0.32
	Treated	5.33 ± 0.34*	5.34 ± 0.79*	4.87 ± 0.42	6.14 ± 0.11*	5.85 ± 0.26*
ACh	Control	7.19 ± 0.81	5.05 ± 0.33	7.37 ± 0.29	8.42 ± 2.01	5.78 ± 0.78
	Treated	6.51 ± 1.87*	4.03 ± 0.02*	6.49 ± 1.62*	5.71 ± 0.63*	4.23 ± 0.13*

Results of the effect of kola nut on the state of oxidation, neurogenesis, and neuronal synapses on the treated and control animals on PND 0, 7, 21, 56, and 70. The data are represented by mean ± SE, with 10 animals in each group. * *p* < 0.5 indicates significant differences between the two groups. *MDA *malondialdehyde, *BDNF *brain-derived neurotrophic factor, *ACh *acetylcholine

Likewise, the level of hippocampal BDNF was significantly different in the treated animals when compared with their respective controls across all age groups, as shown by mean effects of treatment (F _[4,30] =11.70_; *p* = 0.0001), mean effects of age (F _[1,30] =73.55_; *p* = 0.0001), and interaction (F _[4,30] =5.229_; *p* = 0.0026).

The level of ACh was significantly affected in the hippocampus of the kola nut treated animals across age groups, mean effects of treatment (F _[4,30] =15.61_; *p* = 0.0001), mean effects of age (F _[1,30] =8.438_; *p* = 0.0068) and, interaction (F _[4,30] =1.196_; *p* = 0.3327).

### Association between the behavioral tests and biochemical analyses

As it has been established that oxidative stress is one of the key factors causing decline in cognitive and memory impairments, it is of necessity to measure the level of its precision marker, MDA in this present study. MDA is recognized as the commonly used marker for oxidative stress. Likewise, BDNF, a neurotrophin, that is also involved in cognition level was measured. Acetylcholine, which has been associated with normal and abnormal cognitive functions was also measured. The levels of these correlated alongside the behavioral tests results to further substantiate the effects of kola nuts on behavior. This association was investigated using logistic regression analysis. The behavioral correlates of MDA, BDNF, and ACH were obtained. A significant association and a positive correlation were found between the SRT and MDA on PND 7 (OR = 0.99, 95% CI= 0.74 – 0.99, *P* = 0.0059) and rearing and MDA on PND 56 (OR = 0.99, 95% CI = 0.85 – 0.99, *P* = 0.0032). Also, a significant association but a negative correlation was found between NOL and MDA on PND 56 (OR = 0.97, 95% CI = -0.99 – -0.41, *P* = 0.0164); NOR and MDA on PND 21 (OR = 0.90, 95% CI = -0.13 – 0.99, *P* = 0.0502); and RAM and BDNF on PND 56 (OR = 0.90, 95% CI = -0.12– 0.99, *P* = 0.0491). Even though our study did not show a positive association between the reduced memory indicators of the behavioral tests and the level of ACh, it is still of note that the level of ACh was reduced in the kola nut-exposed animals (Table 5).

## Discussion

Ethnomedicine is currently gaining significant traction and attention worldwide because of its efficacy, potency, availability, and low toxicity rate when taken moderately. Meanwhile, few studies have reported the harmful impact of this plant-based treatment (plant natural products) extract on the different organs of the body. Several reports focused only on the phytochemical constituents of these plant-based natural products, rather than looking at the harmful responses they might be stimulating. The brain, a complex organ involved in numerous tasking activities, is vulnerable to free radical damage and decreased functionality, and the hippocampus, a co-regulator, along with the other brain regions responsible for processing memory and learning and cognitive functions are not exempted from this (Guitart-Masip et al. 2010; Lisman et al. 2017).

This study evaluated the effect of kola nuts on spatial and non-spatial memory behavior, spontaneous locomotor reflex behavior, and anxiety and explorative behavior in rats, using CA, SR, spontaneous movement, open field, object recognition and location, and RAM test models. Previous studies have reported that either neurochemical and structural deregulation or metabolic disturbances are implicated in the disruption of neurocellular activities in the juvenile/pups post-birth, and these disturbances could be attributed to the exposure to environmental toxins, drugs, or any other form of stressors (Khazen et al. 2019). Ahmadalipour and Rashidy-Pour (2015) reported that pre-natal exposure to morphine throughout pregnancy results in prolonged or permanent neurochemical and behavioral impairment, including a reduction in learning and memory behavior in children of addicted mothers. Studies have also uncovered that exposure to stressful environmental or traumatic events during the gestation period exacerbates the vulnerability to psychopathology-induced neuroendocrine alterations associated with a decrease in emotion, learning, and memory behavior that appear in the early life stages (Chagas et al. 2021). Interestingly, in our previous study on kola nut extract obtained from *Cola nitida* vent. Schott, we reported that the administration of the extract to pregnant rats induced significant impairment of motor activity and histomorphological alterations in the pups’ cerebellums (Atiba et al. 2021).

However, in the present study, we evaluated the post-natal motor/reflex behavior in pups following intrauterine/gestational exposure to treatment with kola nut (*Cola nitida*) extract. The developmental reflex and memories in the pups at PNDs 4, 5, 6, and 7 were investigated with CA, SR, and spontaneous movement tests, and the data obtained showed that the developmental reflex behavior decreases with increased latency of memory retention when subjected to CA (PND 5, 6, and 7) and SR test (PND 5) a few days after birth. Moreover, on PND 7, the pups showed no further signs of positive behavior, suggesting impaired developmental reflex behaviors and symptoms of hypo locomotion (sluggish movement). These data are further affirmed by the delayed/decreased number of head raising and pivoting. Although, the time taken to pivot reduces as observed in the pups, which could also be attributed to the impaired developmental reflex behavior.

Head raising and pivoting have already been associated with early maturity (Tartaglione et al. 2020); therefore, it is noteworthy that this effect could be due to kola nuts. Thus, delaying the maturity of the treated pups as reported by the reduction in the developmental reflex behavior in this study. The developmental reflexes and memory function were expected to improve in the pups with age, but this was not observed in the kola nut-treated pups. To affirm it further, these findings of the decreased developmental reflex activity and memory retention ability remained present in the animals at an advanced age (on PND 21 and 56). The data showed no signs of increased locomotion, as depicted by no change in mean velocity and the total distance covered.

Moreover, the kola nut-treated animals at an advanced age showed no marked freezing episode but exhibited immoderate grooming behavior, defecation, and urination—indicators of explorative activity reduction and anxiety-like behavior. Grooming, which is primarily used for hygienic purposes can also be used to study stress, anxiety, fear, and cognitive functions. In the present study, kola nut-treated rats had more bouts of grooming than their control counterparts. The increased number of grooming could either be due to fear or inability to remember the environment like it was seen in PND 56. This is in line with the findings of Kalueff et al. (2007), indicating that grooming frequency and exploratory activities could be increased in the presence of stress, anxiety, fear (Smoljnsky et al. 2009) and increase in response to novelty (Pisula et al. 2006).

Also, rearing activity increased in the kola nut-treated animals. This activity is regarded as maladaptive behavior due to the failure of the pups to retain previous memory or consolidate the fact that they were previously present in that environment. These responses could mean that pre-natal kola nut consumption provokes a decline in cognitive function and memory in the pups, possibly at an advanced age. This is in line with the reported findings of Lever et al. (2006) that the number of rearing should reduce as the environment becomes familiar. However, this was not so in our kola nut-treated rats.

The burden of negative cognitive phenotypes, such as amnesia, was also observed in this study. The gestational treatments with kola nut (*Cola nitida*) resulted in irregular retention of previous memory or unsustainable learning and memory functions due to the reduced amount of residual knowledge of the familiar object in NOR and NOL. Moreover, the radial arm maze assessment followed a similar paradigm with irregular or decreased spatial memory function in the pups. Perhaps, the consumption of kola nuts prenatally affected the development and behavioral index for cognitive and spatial memory in this study and tilted more to the deleterious effects of kola nut when consumed in pregnancy. A similar observation was reported by Atiba et al. (2021), indicating that the consumption of kola nut in pregnant animals is detrimental to the developing cerebellar neurons, which might be implicated in spatial memory and motor/cognitive impairment.

The brain is made up of enzymatic and non-enzymatic antioxidants. The antioxidant defense system acts as the first line of defense against the insult of free radicals and reactive oxygen species generated in the system, acquired through the toxic substances consumed, or to which the system has been exposed. The antioxidant defense system mitigates the deleterious effect of reactive oxygen species, thereby averting the cellular oxidative damage causing structural and functional alterations (Zhang et al. 2016; Farella et al. 2021; Onasanwo et al. 2021; Adebayo et al. 2022). In this study, the induction of oxidative stress in the hippocampal tissue was observed across all kola nut-treated pups through the increase of MDA concentration. Joshua et al. (2017) and Erukainure et al. (2017) observed a decrease in the MDA levels in the liver of the rats exposed to H_2_O and treated with kola nut extract. These changes in the MDA levels could be attributed to dose-dependent and tissue-specific differences as a higher dose was used in this study. Notably, the brain is highly susceptible to free radical damage resulting from the excessive accumulation of reactive oxygen species. This induces and exacerbates mitochondrial damage, likewise seen in lipid peroxidation, apoptosis, and inflammation (Kang and Yang 2020). An increase in the MDA level in the hippocampal tissue could depict the probable pro-oxidant role of the kola nut in the tissue, thereby subjecting it to lipid peroxidation and possible tissue damage. The increase in pro-oxidant activity observed in the hippocampal tissues of the kola nut-treated pups could bring about the decreased developmental reflex activity and impaired memory retention ability recorded in this study.

The brain functionality and adaptive capacity are regulated by synaptic plasticity. This is mediated by neurotrophins, which play crucial roles in controlling synapses, the cholinergic system, and neurotrophic factors (e.g., BDNF) in the adult brain (Rozov et al. 2017; Isaeva et al. 2018). The BDNF and the cholinergic system neurotransmitter are broadly distributed throughout the brain, and their expressions are most prominent in hippocampal activities. These are notably cognition, neuroplasticity, synaptic function, and dendritic restructuring (Colucci-D’amato et al. 2020; Ben-Azu et al. 2022). Moreover, a reduction in the BDNF and ACh levels in the hippocampus might impede these processes, making neuroplasticity, memory retrieval and consolidation becoming difficult.

In the present study, the hippocampal BDNF concentration in the pre-natal kola nut-treated animals was minimally elevated. The increased hippocampal BDNF concentration may indicate its adaptive response in the presence of oxidative burden, in order to reverse the decreased developmental reflex behavior and impaired memory and cognitive function in animals (Zhang et al. 2016). This process could upregulate the antioxidant enzymes and repairs of damaged DNA in the hippocampal neurons. Additionally, the hippocampal BDNF expression could be attributed to the caffeine present in kola nuts, which functions in part as a development suppressant, as reported in our previous study (Atiba et al. 2021).

Corroborating this effect, the activity of the cholinergic system decreases with a decrease in ACh release in the animals’ hippocampi. In our study, a steady decrease in ACh concentration was observed in the kola nut-treated pups, inciting a decrease in exploratory novelty in NOR and NOL tests. This was unlike what Giovannini and his colleagues (Giovannini et al. 2001) found that hippocampal ACh increases in the presence of novelty, thereby, increasing exploratory behavior.

Thiel et al (1998) found that ACh level increases in the presence of exploratory rearing compared to non-novel environment or activity. This was contrary to our findings in which increased rearing showed decrease in ACh level in the treated pups and decrease in rearing had increase level of ACh in the control. Maurer and Williams’ (2017) study also reported that a decrease in ACh release affects cognitive memory indices. We may, therefore, conclude that the adaptive production of the BDNF protein, decreased activity of the cholinergic system, and increased redox status in the kola nut-treated animals’ hippocampi may be accountable for the reduced developmental reflex behavior and impaired memory and cognitive function observed in this study.

The biochemical indices were selected to reflect different parameters that do play roles during oxidative stress (MDA), followed by the recovery process and mechanism (BDNF) and the functional state of cognition during memory and learning formation (ACh). These three parameters did provide information that could be used to deduce any potential changes in the hippocampus in this study.

## Conclusion

This study has shown that pre-natal kola nuts consumption is deleterious to the developing brain especially the developing hippocampus. This was portrayed in excessive grooming and rearing, and inability to remember an activity undertaken nor the environment been to. This could impair neuronal plasticity and the cholinergic system, thus causing a delay in developmental reflex behavior and cognitive memory indices. The adaptive response of the biochemical markers investigated do allude to the correlation with factors needed in the neuronal plasticity associated with memory formation in the hippocampus. Further investigation is suggested to elucidate the other mechanism of action through which kola nut and it constituents produce these postnatal effects.

## Data Availability

The datasets generated and/or analyzed during the current study will be available from the corresponding author upon reasonable request.
